# Spatiotemporal variation of nitrate concentrations in soil and groundwater of an intensely polluted agricultural area

**DOI:** 10.1038/s41598-021-82188-2

**Published:** 2021-01-28

**Authors:** Kei Nakagawa, Hiroki Amano, Magnus Persson, Ronny Berndtsson

**Affiliations:** 1 Institute of Integrated Science and Technology, Nagasaki University, 1-14 Bunkyo-machi, Nagasaki, 852-8521 Japan; 2 Department of Kyushu Liberal Arts Education, Tokai University, 9-1-1 Toroku, Higashi-ku, Kumamoto, 862-8652 Japan; 3 Division of Water Resources Engineering, Lund University, Box 118, 221 00 Lund, Sweden; 4 Division of Water Resources Engineering, Centre for Advanced Middle Eastern Studies, Lund University, Box 118, 221 00 Lund, Sweden

**Keywords:** Environmental sciences, Hydrology

## Abstract

Nitrate pollution in groundwater is a serious problem in many parts of the world. However, due to the diffuse and common spatially over-lapping character of potential several non-point pollution sources, it is often difficult to distinguish main nitrate sources responsible for the pollution. For this purpose, we present a novel methodology applied to groundwater for an intensely polluted area. Groundwater samples were collected monthly from April 2017 to March 2018 in Shimabara City, Nagasaki, Japan. Soil samples were collected seasonally at soil surface and 50 cm depth at 10 locations during the same period. Sequential extraction by water and extract agents was performed using calcium phosphate for anions and strontium chloride for cations. Mean nitrate concentration in groundwater close to a livestock waste disposal site (hereinafter called “LWDS”) was 14.2 mg L^−1^, which is exceeding Japanese drinking water standards (10 mg L^−1^). We used coprostanol concentration, which is a fecal pollution indicator, to identify pollution sources related to livestock waste. For this purpose, we measured coprostanol (5β) and cholestanol (5α) and then calculated the sterol ratio (5β/(5β + 5α)). The ratios for three groundwater sampling sites were 0.28, 0.26, and 0.10, respectively. The sterol ratios indicated no pollution (< 0.3). However, the detection of coprostanol originating from animal and human waste showed that groundwater was clearly affected by this pollution source. Nitrate levels in the soil were relatively high in samples collected close to the LWDS and coprostanol contents were affected by livestock waste. Soil and groundwater nitrate concentrations displayed a complex but strong relationship. Nitrate contents were shown to be transported downstream from source areas in both soil and groundwater.

## Introduction

Groundwater polluted by nitrate is a common problem leading to negative human health effects. World Health Organization recommends a maximum of 50 mg L^−1^ of nitrate (NO_3_^−^) in drinking water^1^. In general, nitrate pollution in groundwater is often related to animal husbandry and heavy use of soil fertilizers. To improve the understanding of the fate and transport of nitrate and possible remediation measures, research is needed on new methods to distinguish different spatiotemporal nitrate sources to soil and groundwater. Many studies have focused on nitrate contents in groundwater^2–8^ and soil^5,6,9–13^. Modeling studies indicate that groundwater vulnerability to nitrate pollution can be explained by a complex set of different hydrogeologic variables such as depth to water table, net recharge, aquifer and soil media, topography, impact of the vadose zone, and hydraulic conductivity^14^. Besides this, the spatiotemporal variability of potential pollutant sources creates a second superimposed complexity. In practical applications it is therefore, difficult to distinguish fate and transport properties of nitrate from different overlapping pollutant sources. Similarly, potential remediation techniques are hampered by this lack of knowledge. Thus, it is important to develop new methods that can be tested on various experimental areas and observations of nitrate concentrations combined with transport modeling to efficiently evaluate future environmental effects and potential remediation alternatives^15,16^.

Shimabara City, Nagasaki, Japan, depends solely on groundwater for its public water supply. The city has been monitoring nitrate-nitrogen (NO_3_-N) concentration in public water wells since 1975. In general, NO_3_–N levels in the wells have continuously increased over the years. Recently, the NO_3_-N concentrations exceeded the Japanese drinking water standard of 10 mg L^−1^ in some wells. High nitrate levels in groundwater have been observed especially in the northern parts of the city for large groundwater depths (> 50 m)^17,18^. High nitrate concentrations in surface water have also been reported^19^. In the northern parts of the city, the potential nitrate load was estimated in 1975 at about 258 kg N year^−1^ ha^−1^. This figure increased to about 308 kg N year^−1^ ha^−1^ in 1985, then reached 436 kg N year^−1^ ha^−1^ in 1995. In 2010, the load was estimated at 455 kg N year^−1^ ha^−1^, which is 1.8 times that of 1975^20^.

As mentioned above, high nitrate concentration in drinking water can affect human health and for infants there is an elevated risk of methemoglobinemia disease. Even though nitrate concentrations may not pose a fatal threat to humans, they do indicate the possible presence of other serious agricultural contaminants. Nitrate pollution is often related to agricultural activities and runoff from fertilizers or leachate from animal husbandry^17–19^. For this reason, evaluation of nitrate leaching from farmlands to surrounding water bodies and the environmental capacity of soils need to be assessed. It is evident that high nitrate concentration in soils is related to corresponding concentrations in surface and groundwater. However, these relationships are complex and not easy to quantify. Thus, although there have been previous studies on the nitrate in surface water and groundwater^17–19^, joint studies including soil nitrate and assessment of pollutant sources are still few.

Identifying the source of nitrate is a first step to unravel the complex relationships among groundwater, surface water, and soil. Previous studies have indicated that the spatial distribution of nitrate concentration in both surface and groundwater in Shimabara City is associated with the use of chemical fertilizers and livestock waste^17,19^. To identify individual nitrate pollution sources, isotopic nitrate data have proven successful^21–24^. However, some observations may not have a clear isotopic signature and not a clear association with a certain pollutant source^21^. To overcome this problem, coprostanol can be used to indicate fecal pollution and to distinguish between dominant nitrate pollution sources in groundwater^25^. Considering that the coprostanol content in groundwater is often quite low due to adsorption to soil and rock material, special analyses techniques are needed^26^. For this purpose, the sterol ratio between coprostanol and cholestanol may as well be investigated to indicate dominant nitrate pollution sources^26^. Coprostanol and sterol ratio are thus, parts of a comprehensive methodology that can be used to study the complex spatiotemporal nitrate variation and interactions between soil and groundwater. To the authors´ knowledge, comprehensive studies on this are still rare in the research literature.

In view of the above, the main objective of this study is to present and test a novel methodology that can be used to improve the knowledge on the fate and transport of nitrate pollutants in complex and heavily polluted soils and groundwater. For this purpose, we investigated coprostanol content in soil, and sterol ratio in groundwater in the study area. A further objective was to investigate the relationships between soil, surface water, and groundwater in terms of nitrate concentration and to improve the understanding of nitrate dynamics in the area. To accomplish these objectives, we collected soil and groundwater samples close to a previously used livestock waste disposal site (LWDS) that was in use from 2000 and from downstream areas. We then used data obtained from previous studies^17,19^ to assess the nitrate dynamics of the area in soil and water since the LWDS is a possible main source of pollution. The LWDS has been in use during a prolonged period from 2000 to 2006 and nitrate is likely to remain in the soil due to slow natural attenuation, affecting both surface and groundwater downstream of the site. Thus, there is a possibility to monitor nitrate pollutants from the LWDS site using coprostanol as a tracer and map mixing and transport dynamics in the soil and water phase. The suggested novel methodology can potentially give us new insights of nitrate pollution fate and transport in the study area and new and improved knowledge on nitrate dynamics.

### Study area

**Figure 1:**
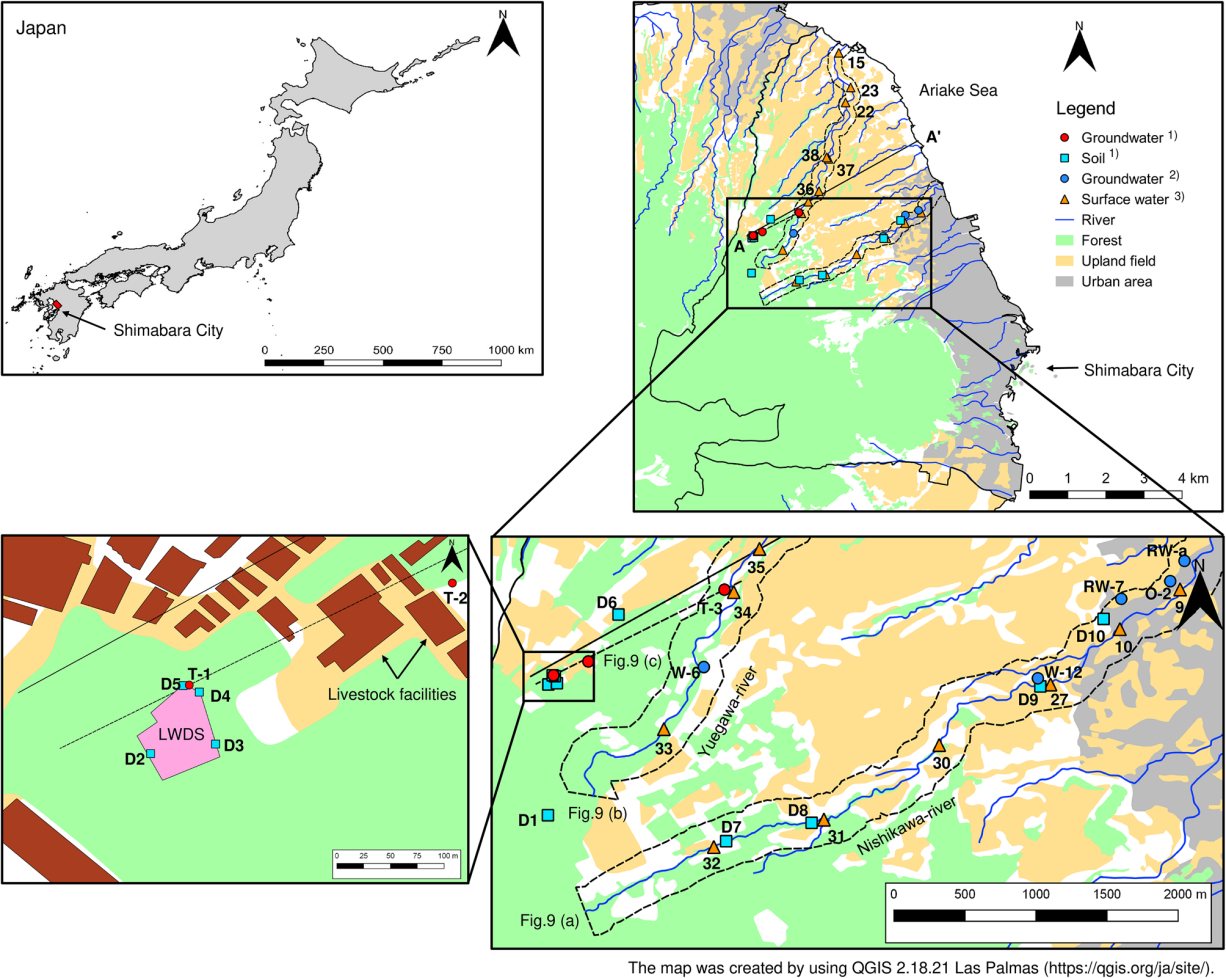
Study area. (1) this study. (2) Nakagawa et al.^17^, (3) Amano et al.^19^.

Shimabara City is located in the northeast part of the Shimabara Peninsula, Nagasaki, Japan (Fig. 1). The area of Shimabara City is 82.8 km^2^, occupying 18% of the Shimabara Peninsula. Shimabara City lies on the alluvial fan that spreads as a gentle slope from the Mt. Fugendake in the center of Shimabara Peninsula. The geology is formed by volcanic deposits composed of andesite, dacite, and tuff breccia. Upland agricultural areas are concentrated to the northern part of Shimabara City (Fig. 1). The cultivation area is 18.5 km^2^ and comprises 22.3% of Shimabara City (2015). The livestock raised in the area is about 1000 milk cattle, 23,000 pig, and 1,030,000 chicken (2015). Many livestock raising facilities are located in the upstream areas of the Nishikawa and Yuegawa Rivers. The study area has a humid subtropical climate with an annual mean temperature of 17.1 °C^27^. The area received 1989 mm of precipitation in 2017. Normally, the rainy season occurs during June and July and precipitation amount was 571.5 mm (2017) (28% of annual amount that was less than usual).

**Figure 2:**
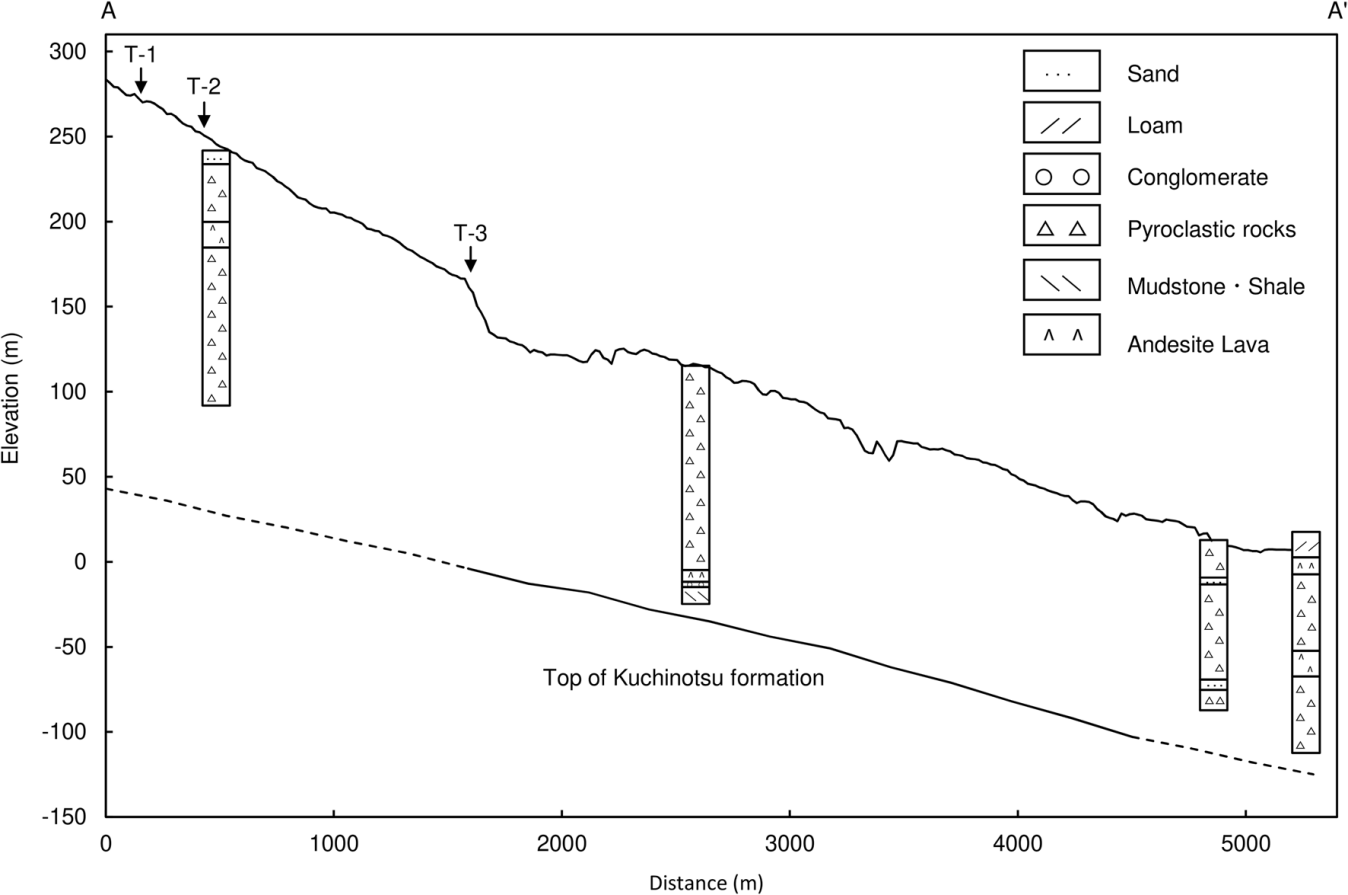
Geological cross section of line A-A’ in Fig. 1.

The hydrogeology of the study area has been described by Murakami^28^. Figure 2 shows a geological cross section according to the A-A’ line in Fig. 1. The figure was constructed using geological profiles from boreholes^28^. The northern parts of the peninsula present a gentle slope that form an alluvial fan from the Mt. Fugendake composed of volcanic rock. The basement rock (Kuchinotsu Layer), has a circular arc-shaped structure that is gently inclined toward the coast. Close to the coast, the basement rock is distributed between 10 and 100 m below mean sea level. A thick layer of Paleogene period shale is found in the lower part of the Kuchinotsu Layer (360 to 420 m below mean sea level). Rock on top of the Kuchinotsu Layer forming a foot of the mountain is called Pre-Unzen volcanic rock (Tatsuishi Layer). This consists of tuff breccia, tuff, and volcanic conglomerate. The thickness of this layer is 100–150 m, and the top parts are constituted of weathered rock and sand gravel layer, which forms an unconfined aquifer^29^. Soil of the lower parts contains a confined groundwater aquifer. In the layer above the Tatsuishi Layer, the groundwater is unconfined^29^. Above 300 m amsl, Unzen volcanic rock forms the mountain area. This mainly consists of hornblende-andesite that displays mature joints and splits. The Unzen volcanic rock is favorable for large groundwater recharge. Near the boundary with Pre-Unzen volcanic rock, groundwater discharges as springs to the soil surface. Alluvium deposits have mainly developed in limited areas of the lowland in the river basin. The central parts of the peninsula belong to a subsidence area called Unzen Graben. In these central parts, active volcanos such as the Mt. Fugendake are composed of hornblende-andesite and dacite that form steep mountain areas. As mentioned above, the Pre-Unzen volcanic rock is extensive and forms the major groundwater aquifer in Nagasaki Prefecture. Debris from the collapsed Mt. Mayuyama, located west of Shimabara city, is distributed in the urban area. Rainfall over this area infiltrates into deposits and becomes unconfined/confined groundwater that subsequently flows toward the Ariake Sea. Thus, artesian wells can be found close to the coastal line.

## Materials and methods

### Sampling and analysis

Sampling locations are shown in Fig. 1. To assess nitrate pollution sources, groundwater samples were collected once a month from three wells (T-1, T-2, and T-3) close to a livestock waste disposal site (LWDS) and the downstream area from April 2017 to March 2018. The depth of these wells is about 50 m from soil surface. Screens were installed in all wells. The groundwater in T-1 and T-2 are unconfined. On the other hand, as T-3 is an artesian well, it probably is connected to the lower parts of the Tatsuishi Layer. Soil samples were collected seasonally at soil surface and 50 cm depth at 10 locations. These locations included the LWDS that was used for secondary treatment of liquid manure (D2–D5), downstream of LWDS (D6), Nishikawa River Basin (D7–D10), and forest (D1) during the same period. The area of the LWDS is about 2000 m^2^, and its location is upstream in the Yuegawa River Basin (Fig. 1). Sampling around the LWDS was performed due to that the site is expected to be a main nitrate pollution source. For this reason, as well, soil samples were collected downstream of the LWDS (upland field). Two rivers (Yuegawa and Nishikawa River) and groundwater in this basin are heavily polluted by nitrate with a highest observed level of 27.5 mg L^−1,^^17,19^. We also collected soil samples from the Nishikawa River Basin as a comparison to Yuegawa River Basin. The LWDS was constructed by digging a 50 cm trench in the soil, installing a drainage pipe at a soil depth of 35 cm, and then backfilling the trench. At time of construction, it was expected that most of the manure would be absorbed by the soil and excess water would be eliminated by evaporation and drainage. Thus, soil samples were collected from different soil depths. Sampling location T-1 is situated between the LWDS and the treatment plant for liquid waste. Operation of the treatment plant started in 2000 just after the Japanese livestock waste disposal law changed (1999). The LWDS was taken into use in 2000 to constitute a second treatment step added to the original treatment plant. Use of the LWDS was halted in 2006 because the treatment capacity of the plant was improved. Today, the effluent from the plant meets the standard 600 mg L^−1^ of treated waste water, but may exceed drinking water standard.

Portable meters (HORIBA D-51 and D-54) were used for measurements of pH, oxidation–reduction potential (ORP), and electrical conductivity (EC). Dissolved oxygen (DO) was measured with a luminescence-based sensor (HACH HQ30d). HCO_3_^−^ was quantified using titration with 0.1 N HCl. These variables and water temperature were measured on-site. Sequential extraction by water and extract agents was performed for the soil samples. As extract agents, firstly water for both cations and anions, then calcium phosphate for anions and strontium chloride for cations were used. The major cations and anions (Na^+^, NH_4_^+^, K^+^, Mg^2+^, Ca^2+^, F^−^, Cl^−^, NO_2_^−^, NO_3_^−^, and SO_4_^2−^) found in the water and extract samples were analyzed by ion chromatography (Metrohm 861 Advanced Compact IC). As well, coprostanol and cholestenol concentrations were monitored in the groundwater as indicators of fecal pollution. Sterols were extracted from all samples by liquid–liquid extraction with water-dichloromethane. After dehydration and concentration, the extract was converted to trimethylsilyl using bis (trimethylsilyl) trifluoroacetamide (BSTFA) followed by quantification utilizing gas chromatography-mass spectroscopy (GC-MS)^25^. A more detailed description of the analysis method for sterols is described in Nakagawa et al.^26^.

### Data analysis

To understand the temporal fluctuation of nitrate concentration, it is important to quantify precipitation events. Previous studies have shown that nitrate concentrations are clearly diluted after precipitation events^17,18^. Following previous studies, the relationship between precipitation and nitrate in groundwater and soil was evaluated using sterols as tracers. Precipitation data from Shimabara Observatory were downloaded from the Japan Meteorological Agency Website^27^.

We used coprostanol concentration as a tracer and to identify pollution sources related to livestock waste^25,26^. Elevated coprostanol concentration is a fecal pollution indicator. We measured coprostanol (5β) and cholestanol (5α) and then calculated the sterol ratio (5β/(5β + 5α)). The sterol ratio was used as an indicator to evaluate fecal pollution at three levels: no pollution, uncertain, and certain pollution^30^. This suggested methodology has not been tested previously.

As mentioned above, the area close to the LWDS is expected to constitute a main pollution source. In order to evaluate a potential risk of nitrate pollution in groundwater from soil, nitrate concentrations in soil were converted to groundwater nitrate concentrations using a following regression equation suggested by Wang et al.^9^:

1 $$N_{g} = 0.058N_{s} + 1.17$$

where *N*_*g*_ is nitrate concentration (mg L^−1^) in groundwater, and *N*_*s*_ is nitrate concentration (mg kg^−1^) in soil.

To apply appropriate countermeasures against nitrate pollution, careful site examination is needed, thereby evaluating the relationship between soil, groundwater, and surface water. Finally, nitrate transport relationship between soil, surface water, and groundwater along the two polluted Nishikawa and Yuegawa rivers, was evaluated by projecting soil, surface water, and groundwater data vertically along the stream length within 200 m from the mainstream river according to the plotted dot-line area in Fig. 1. The nitrate concentrations in water and soil were projected to the straight line from T-1 to T-3 (Fig. 1). Spatial variation of nitrate concentrations in groundwater had a similar pattern as the corresponding surface water, even though groundwater samples showed slightly larger concentrations^17,19^. In addition to the groundwater data that we obtained in this study, groundwater data from a previous study representing mean of observations from 2011 to 2013^17^ were used. Surface water data were studied during 2017^19^ at 42 locations. In the total 13 samples were collected from Yuegawa river including tributaries. In Nishikawa river, six samples were collected. Most of the water samples were collected directly from the center sections of the rivers.

## Results

### Groundwater

**Figure 3:**
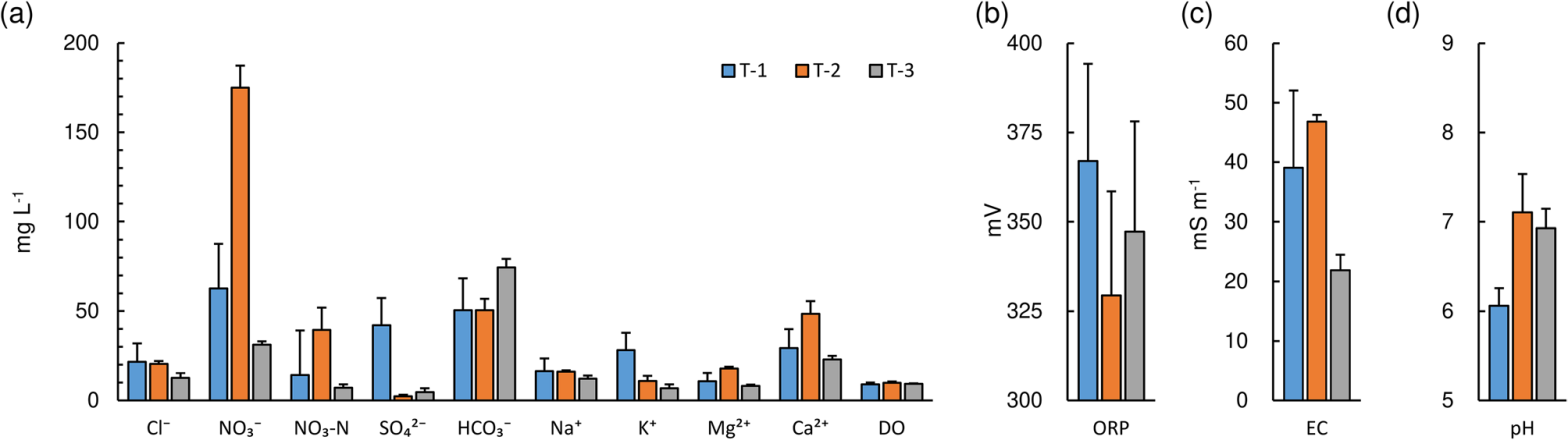
Chemistry of groundwater (**a**) Major ion components and DO, (**b**) ORP, (**c**) EC, and (**d**) pH.

Measured hydrochemical variables in the groundwater are summarized in Fig. 3. In addition, all analyses results for each well can be found in Supplementary Table S1. Ca^2+^ was the dominant cation for all sampling sites. Water samples at T-1 and T-2 were dominated by NO_3_^−^ while HCO_3_^−^ was dominant at T-3. This shows that the chemical composition of groundwater in the area is Ca–NO_3_ and Ca–HCO_3_ type. Although, T-3 had a water chemistry of Ca–HCO_3_ common to most shallow groundwater in Japan^31^, the other sites had unusual contents of some ions according to the below. T-1 was characterized by K^+^ with a mean concentration of 28.1 mg L^−1^. This site had the highest average Cl^−^ corresponding to 21.6 mg L^−1^ for the three sites. T-2 had significantly higher NO_3_–N with an average concentration of 39.5 mg L^−1^. The average DO range from 9.0 to 9.8 mg L^−1^. All DO concentrations were above 5.0 mg L^−1^ indicating small pollution from organic matter^32^. The average ORP represented positive values from 329.4 to 367.0 mV, indicating that the groundwater is in an oxidant state. The average EC ranged from 21.9 to 46.8 mS m^−1^, reflecting the amount of dissolved ions. The average pH was between 6.1 and 7.1, representing slightly acidic to neutral conditions.

**Figure 4:**
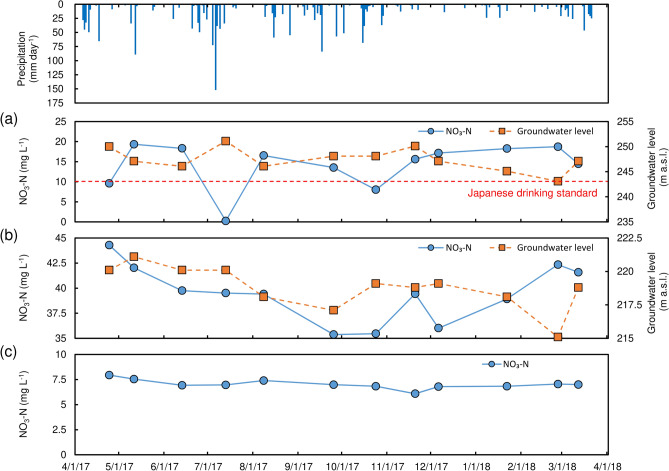
Temporal variation of precipitation, nitrate concentration in groundwater, and groundwater level (**a**) T-1, (**b**) T-2, (**c**) T-3.

Temporal variation in precipitation, nitrate (NO_3_–N) concentration, and groundwater level are shown in Fig. 4. Mean nitrate (NO_3_–N) concentration in groundwater at T-1 was 14.2 mg L^−1^, which is exceeding Japanese drinking water standards (10 mg L^−1^). After precipitation events, a general decline in nitrate concentration was observed. A similar tendency can be seen at T-2. Mean nitrate concentration in groundwater at T-2 was 39.5 mg L^−1^, which is considerably exceeding Japanese drinking water standard. Mean nitrate concentration in groundwater at T-3 was 7.0 mg L^−1^, which is below drinking water standards, but still represents a relatively high concentration.

**Figure 5:**
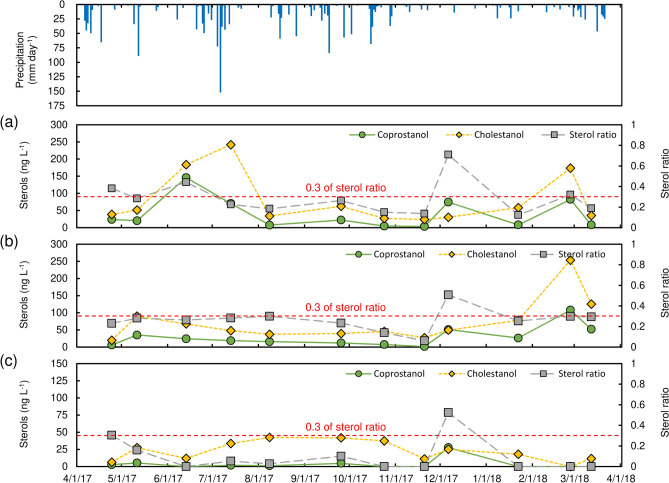
Temporal variation of coprostanol, cholestanol, and sterol ratio in groundwater (**a**) T-1, (**b**) T-2, (**c**) T-3.

Temporal variation in coprostanol, cholestanol, and sterol ratio are shown in Fig. 5. The mean ratios at T-1, T-2, and T-3 were 0.28, 0.26, and 0.10, respectively. According to proposed criteria of sterol ratio, these values indicate “no pollution” (< 0.3)^30^. However, samples at all locations in December showed high sterol ratio (0.51–0.71). Samples at T-1 exceeded this criterion four times. Thus, this site is obviously affected by livestock waste. The mean concentration of coprostanol at T-1, T-2, and T-3 was 39.4, 30.0, and 3.62 ng L^−1^. T-1 and T-2 showed above or close to 30 ng L^−1^. High concentrations of coprostanol and cholestanol were detected in June at T-1, which were 146.0 ng L^−1^ and 184.0 ng L^−1^, respectively.

### Soil

**Figure 6:**
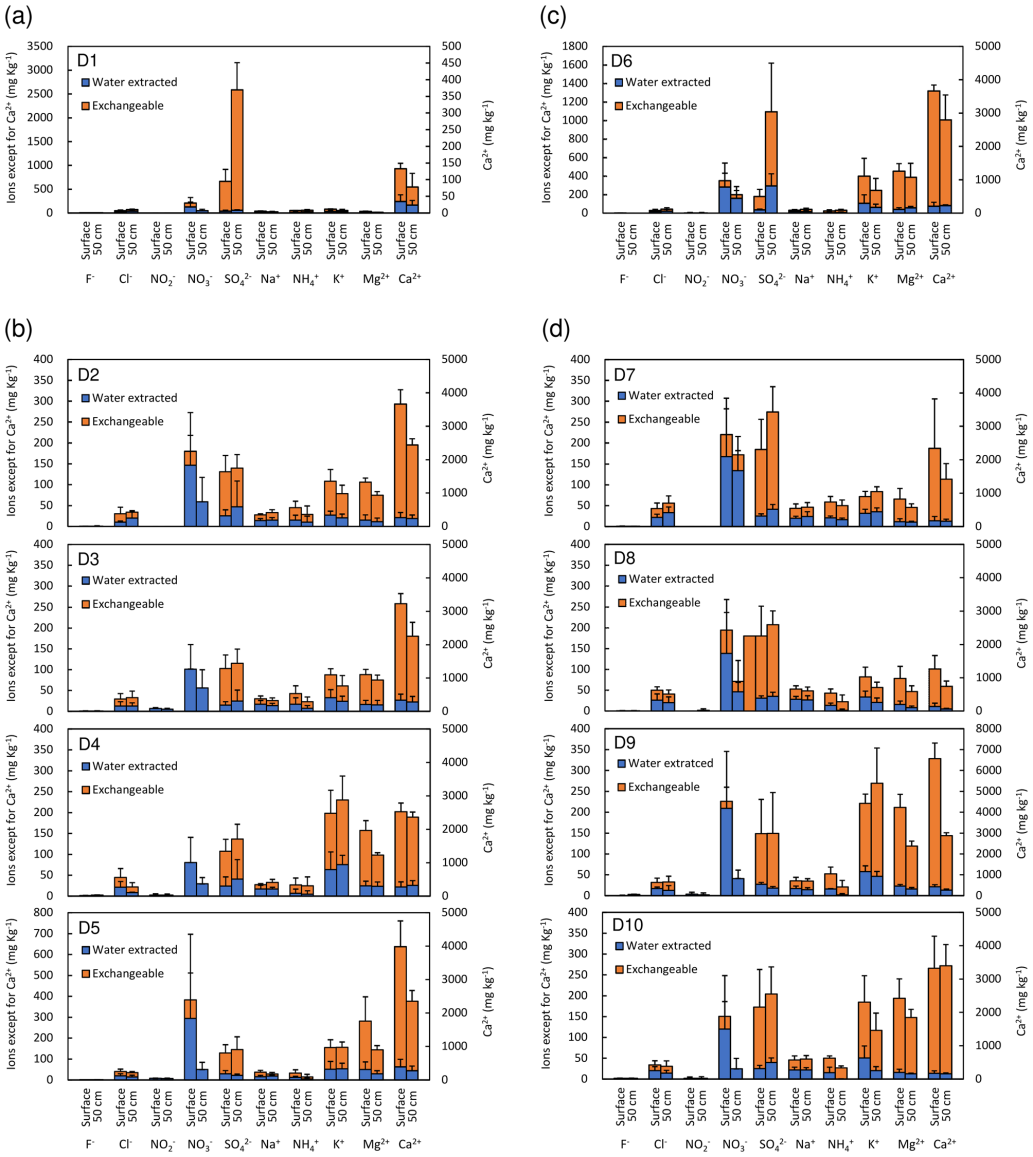
Major ion components in different soil types (**a**) forest, (**b**) LWDS, (**c**) upland field, and (**d**) Nishikawa River basin.

**Figure 7:**
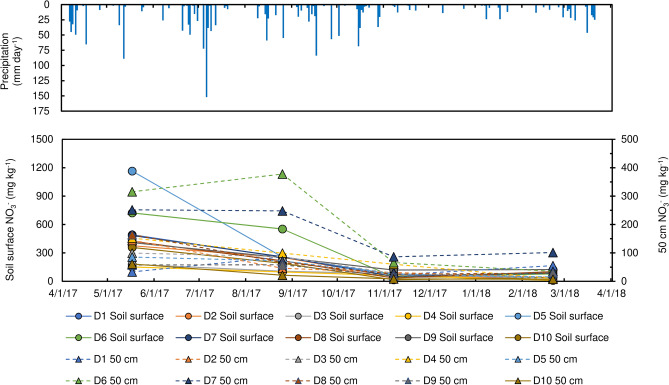
Temporal variation of nitrate in soil.

Major ion concentrations in soil are shown in Fig. 6. The graphs are divided into water extracted and exchangeable fractions. Most of the nitrate ions (NO_3_^−^) are concentrated to the water extracted fraction. This confirms that nitrate ions are easily dissolved in soil water. Adsorbed nitrate concentrations gradually decreased from June to November at all sampling locations due to dilution by precipitation during the rainy season (Fig. 7). In Fig. 7, adsorbed nitrate corresponds to obtained nitrate from the soil by sequential extraction using water and calcium phosphate. Generally, nitrate concentrations at the soil surface are higher than those of the 50 cm soil depth.

Nitrate in the soil is relatively high from 29.4 to 382.7 mg kg^−1^ in samples collected at the LWDS adjacent to the livestock waste treatment plant (D2–D5). Ca^2+^ also shows a high concentration of 2257.4–3984.5 mg kg^−1^ at these sites. Especially at D4, samples have high K^+^ concentration of 230.2 mg kg^−1^ at the 50 cm depth. The concentration of adsorbed nitrate is relatively high (about 200 mg kg^−1^) at the four sampling sites in Nishikawa River Basin (D7–D10). Especially at soil depth 50 cm, nitrate concentration is high in the upstream area (D7: 171.9 mg kg^−1^ and D8: 71.2 mg kg^−1^). The ammonium (NH_4_^+^) concentration of 20.8–59.0 mg kg^−1^ in soil along the Nishikawa River is as high as soil samples from the LWDS. A maximum ammonium concentration of 47.6 mg L^−1^ was detected in the water samples from the Nishikawa River^19^. If all of NH_4_^+^ is transformed to NO_3_^−^, NO_3_^−^ concentration corresponds to 164.0 mg L^−1^. At the downstream (D9 and D10), cations such as K^+^, Mg^2+^, and Ca^2+^ display high concentrations of 117.0–269.2 mg kg^−1^, 118.8–211.6 mg kg^−1^, and 2874.6–6562.9 mg kg^−1^, respectively. In addition, SO_4_^2−^ concentration is also high, ranging from 148.4 to 203.9 mg kg^−1^. Meanwhile, at the upstream (D7 and D8), higher concentration of SO_4_^2−^ with 180.4–274.2 mg kg^−1^ also was present. The soil from the upland agricultural area displayed high nitrate concentration (D6) of 349.8 and 198.64 mg kg^−1^ at each depth. In addition, SO_4_^2−^ concentration of 1094.8 mg kg^−1^ at the 50 cm depth was relatively high. Ca^2+^ showed high values, 3662.0 and 2796.3 mg kg^−1^ at soil surface and 50 cm depth, respectively, similar to the LWDS soil.

**Figure 8:**
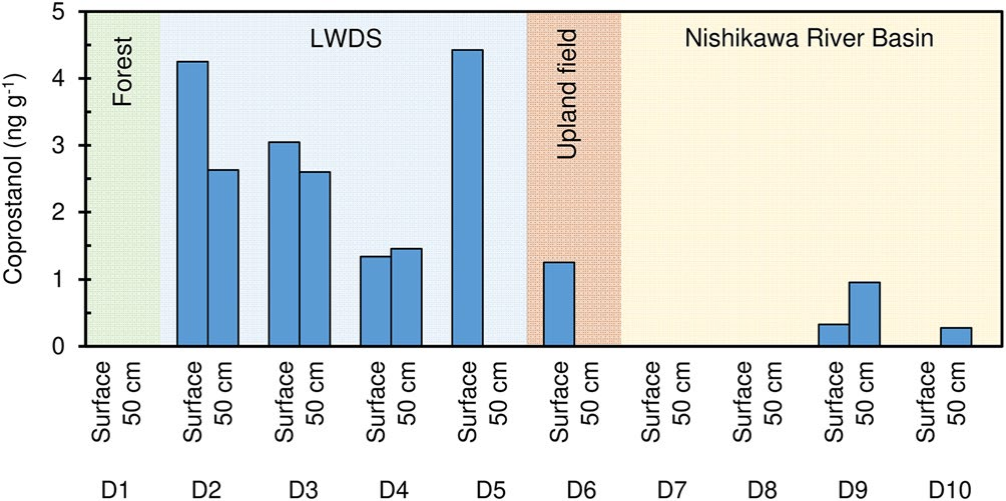
Water soluble coprostanol in soil (May, 2017).

Coprostanol concentrations in water leached soil samples collected in May 2017 are plotted in Fig. 8. The results clearly show that the soil in the LWDS adjacent to the livestock waste treatment plant (D2–D5) has much higher contents as compared to soils in the Nishikawa River Basin (D7–D10). Coprostanol concentration is higher for surface soils as compared to 50 cm depth, similarly as for nitrate concentration.

### Nitrate relationships between soil, surface water, and groundwater

**Figure 9:**
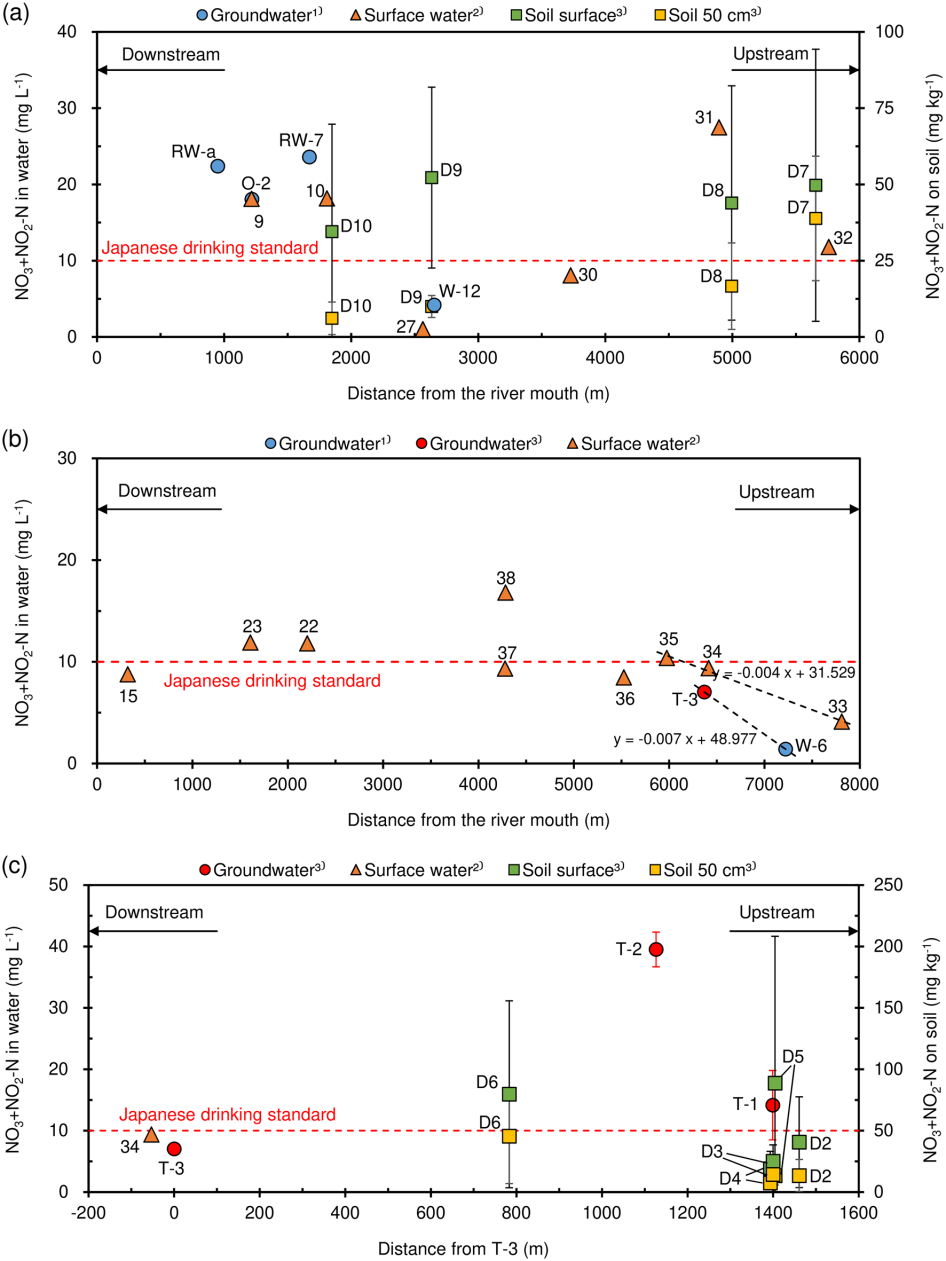
Distribution of nitrate concentration in soil, groundwater, and surface water (**a**) Nishikawa River, (**b**) Yuegawa River, and (**c**) LWDS and downstream. (1) Nakagawa et al.^17^, (2) Amano et al.^19^, (3) this study.

Nitrate concentration in the soil, surface water, and groundwater along the Nishikawa River is summarized in Fig. 9a. From the figure it is seen that there is no clear relationship between soil and water samples. However, the concentration in soil samples (D7 and D8) appears to be related to the surface water concentration in the upstream of the river (> 3500 m from the river mouth). In the case of 50 cm soil depth, it can be seen that there is a slight tendency of higher concentration in the upstream areas. In the upstream (D7), 50 cm depth concentration in soil is high and close to the concentration of the surface soil. The 50 cm depth concentrations then gradually decrease in the downstream direction. Thus, on soil surface, pollutants are easily transported by surface runoff.

**Figure 10:**
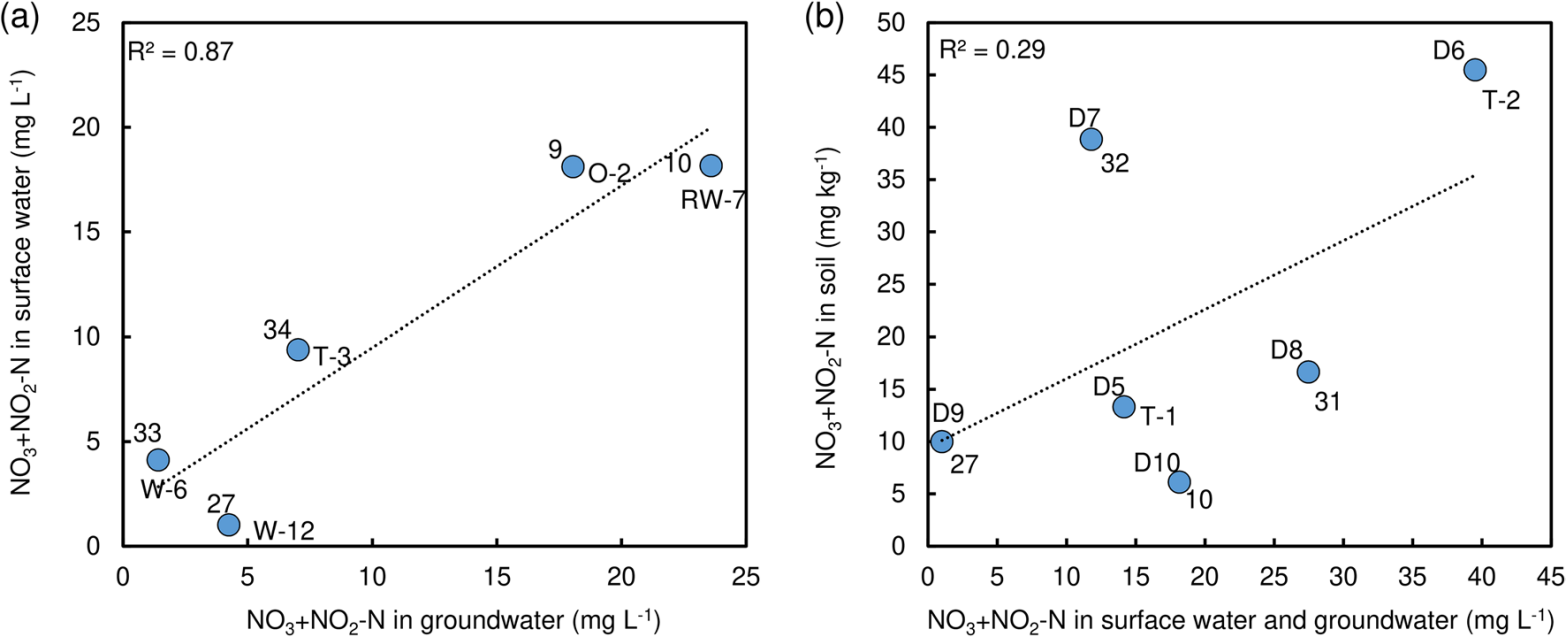
Nitrate concentration relationships for groundwater, surface water, and soil.

Figure 9b shows the relationship between surface and groundwater as both display a high nitrate concentration along the Yuegawa River. According to the negative gradient of the regression equation, a decreasing tendency for both surface and groundwater between 6000 and 7500 m from the river mouth is seen (*y*-axis shows nitrate concentration in water, and *x*-axis shows distance from the river mouth). Except for this upstream region, most of the sampling points along the river show high concentration close to or above Japanese drinking water standards. As shown in Fig. 9, nitrate concentrations in groundwater and surface water clearly show a similar tendency up to 3000 m along the Nishikawa River (Fig. 9a) and 6000 to 8000 m along the Yuegawa River (Fig. 9b). By using data at nearby locations, the correlation between surface and groundwater concentration was calculated (Fig. 10a). Accordingly, a coefficient equal to 0.93 displays a strong connection.

Similarly, nitrate concentration in soil and water is plotted along a line from T-3 to T-1 (Figs. 1 and 9c). T-2 displays a very high concentration in groundwater. This site is located at the downstream of the animal waste treatment plant. Soils contain about twice the concentration of nitrate as compared to other sites in the Nishikawa River Basin. These concentrations do not decrease in the downstream direction. The concentration 7.0 mg L^−1^ in groundwater at T-3 approximately matches the concentration 9.4 mg L^−1^ in surface water just downstream (concentrations are slightly below Japanese drinking water standard). The correlation coefficient between water (groundwater and surface water) and soil content gives 0.54 for 50 cm soil depth (Fig. 10b). This indicates a clear connection between soil and water content regarding nitrate.

## Discussion

### Groundwater

Except for T-3 with expected water chemistry, T-1 and T-2 wells represent unusually high concentration of K^+^, Cl^−^, and NO_3_–N. According to the location of these wells (Fig. 1), it may be assumed that the site is affected by livestock waste from the LWDS and livestock facilities. Not only groundwater but also surface water of Yuegawa River, is severely polluted by nitrate^19^. Previous studies in the area have not revealed such high NO_3_–N concentrations^17^ (range of 25.8 mg L^−1^). The study area has a geological feature consisting of complex pyroclastic sediments on the bedrock as mentioned above. Due to this, flow paths and groundwater recharge patterns are complex potentially following structures of preferential pathways including lava tubes, faults, and fractures. Thus, spatial heterogeneity may be critical factor for nitrate transport and distribution in the study area.

Heavy precipitation corresponding to 344.5 mm during 9 days during the rainy season (July), increased the groundwater level and decreased nitrate concentration by dilution to 0.3 mg L^−1^ in T-1. Also other ion concentrations significantly decreased during this precipitation event from June to July as seen in Supplementary Table S1. There is a positive correlation (*r* = 0.72) between groundwater level rise and precipitation amount two weeks before the sampling date. Consequently, an inverse correlation between groundwater level and nitrate concentration is clearly present at this site. Thus, it is clear that the nitrate concentration was significantly affected by dilution from infiltrating rainfall. However, the diluted concentration was still above 35 mg L^−1^ in T-2. In T-1, the water depth from the soil surface ranged from 27.0 to 35.0 m. On the other hand, the water depth in T-2 ranged from 29.0 to 35.0 m. A shallower groundwater table was usually observed in T-1 as compared to T-2. The depth of water table is an important indicator of groundwater recharge (water table rise) and dilution effect. T-2 receives water also from other livestock facilities (Fig. 1). T-2 is located about 250 m downstream of T-1. Since the sample of T-3 was taken from the confined aquifer, it is not affected by precipitation and thus, shows a relatively stable concentration. The location of T-3 is 1.4 km downstream from T-1.

A sterol ratio above 0.3 means that input of coprostanol into the groundwater is larger than formation of cholestanol by microbial reduction in natural environment. Increased water temperature will promote the activity of microbes^33^. As water temperatures were lowest in December (see Supplementary Table S1), one of the reasons for the high sterol ratio can be effects of microbe activity and introduction of cholestanol for a lower water temperature. 30 ng L^−1^ is a relatively high concentration of coprostanol in groundwater. In total, 22 out of 33 coprostanol concentrations were 20.7–29.6 ng L^−1^ or undetected in a previous study in the area^25^. Recorded high coprostanol concentration of 146.0 ng L^−1^ in June at T-1 (Fig. 1), is obviously related to leachate of treated livestock waste. Onset of the rainy season in June promotes groundwater recharge and leachate formation. In any case, the detection of coprostanol originating from animal and human waste showed that groundwater was clearly affected by this pollution source.

Our proposed method using nitrate concentration and sterol ratio, showed that livestock waste is the main nitrate source in surface water with above 10 mg L^−1^ of nitrate and 0.3 of sterol ratio^26^. Nitrate and sterol ratio tended to be above or near these thresholds at sampling sites (T-1 and T-2) that are affected by livestock waste and high concentrations of K, Cl, and coprostanol. Thus, our method involving sterol ratio can be used to identify complex nitrate sources in groundwater.

### Soil

Most of the nitrate ions were extracted by water. On the other hand, cations and other anions are not easily extracted by water. Thus, it may be confirmed that nitrate is easily dissolved in groundwater due to high water solubility and weak adsorption properties as summarized by Chitsazan et al.^2^, causing water pollution. Common for all sampling sites was that nitrate concentration at the soil surface was higher than at 50 cm depth. The vertical variation may reflect shallow banding of fertilizer, soil mineralization, or net upward transport of nitrate as a result of evaporation^34^. Nakagawa et al.^35^ demonstrated by a series of soil column experiments and numerical simulation that evaporation promotes upward movement of anions.

High nitrate content in soil is beneficial for crop cultivation, however, excess nitrate is easily leached to the groundwater due to high water solubility and weak adsorption properties as mentioned above. Depending on crop type and stage of cultivation, nitrogen demand varies. Thus, it is difficult to define a maximum permissible nitrate content in the soil. Su et al.^10^ estimated that background values of soil nitrate may be about 2.6 mg kg^−1^ for agricultural soils in China. All soil samples in our study area exceeded this value. Nitrate levels in surface soil were input to a regression equation between groundwater and soil nitrate suggested by Wang et al.^9^. As a result, the estimated nitrate (NO_3_–N) level in groundwater is in the range from 1.32 to 5.28 mg L^−1^. However, since 11 years have passed since 2006 and the closure of the LWDS, the plume of high nitrate concentrations is expected to have moved to larger soil depth. This is confirmed by the estimated nitrate concentrations that are below the Japanese drinking water standard of 10 mg L^−1^. Even so, the closed LWDS site is expected to have affected the groundwater.

There are many kinds of fertilizers used in the study area^36^. One fertilizer contains specific components such as (NH_4_)_2_SO_4_, Ca(H_2_PO_4_)_2_·H_2_O + CaSO_4_, and K_2_SO_4_. Another type includes Mg(H_2_PO_4_)_2_, K_2_SiO_3_, NH_4_Cl, and KCl. CaCO_3_ are applied for disinfection of deceased animals in livestock facilities. Livestock waste includes Cl^−^ and essential nutrients such as N, P, and K. Thus, high nitrate, Ca^2+^, and K^+^ concentration in D2-D5 (LWDS) may be related to these factors. At the downstream of Nishikawa River (D9 and D10), high potassium may also be related to the livestock waste. However, Ca^2+^ and Mg^2+^ are related to chemical fertilizers commonly applied together with (NH_4_)_2_SO_4_. Probably, nitrate pollution sources are mixed with chemical fertilizers and livestock waste in the downstream area of the Nishikawa River Basin. Meanwhile, high concentration of SO_4_^2−^ at the upstream (D7 and D8) is also related to application of chemical fertilizers such as an ammonium sulfate, (NH_4_)_2_SO_4_. This may be regarded as a major pollution source in the upstream of Nishikawa River. Relatively high concentration of SO_4_^2−^ and Ca^2+^ at D6 is probably also a result of fertilizer application and CaCO_3_ for disinfection. High SO_4_^2−^ concentrations of 664.3 and 2585.1 mg kg^−1^ in the forest may be naturally occurring in the soil (D1).

The high coprostanol concentrations at D2–D5 (LWDS) are due to disposed livestock waste that risk to leach nitrate to the groundwater. D6 is located in the upland agricultural area close to an animal husbandry farm and it is likely that manure has been applied here as fertilizer. Accordingly, coprostanol was detected in the soil surface at D6. Some threshold values of coprostanol have been suggested to indicate sewage contamination such as 100 ng g^−1^ in river sediments^37^. Accordingly, observed levels in our study indicate no sewage contamination.

### Nitrate relationships between soil, surface water, and groundwater

Pollutants from not only soil but also waste water drained from pig farms affected nitrogen content in the water environment^19^. On the other hand, vertical transport of pollutants is slow (e.g., 0.22–0.26 m year^−1^ for nitrate^38^) that eventually may reach larger soil depths after considerable time. These processes explain the effects of the upstream pollution sources. Even if the nitrate concentration in groundwater is below the Japanese drinking water standard 10 mg L^−1^, the soil has more or less the same high amount of nitrate along the upstream to downstream between 2500 and 4000 m from the river-mouth in Nishikawa River. The annual variation of nitrate concentration in the soil (Fig. 7), indicates a clear leaching tendency with precipitation. In addition, the correlation between water and soil regarding nitrate concentration, suggests risk of future increased nitrate contents in both surface and groundwater.

## Conclusions

Research is needed to improve methods for distinguishing different spatiotemporal nitrate sources to soil and groundwater. The spatiotemporal variability of potential pollutant sources creates a strong complexity that is difficult to decipher using traditional methods. The common soil and geologic media complexity creates a second difficulty in this context. In practical applications it is therefore, difficult to distinguish fate and transport properties of nitrate from different overlapping pollutant sources. Thus, it is important to develop new methods that can be tested on various experimental areas and observations of nitrate concentrations.

In our study, two of three groundwater samples displayed elevated mean nitrate concentration exceeding Japanese drinking water standard (10 mg L^−1^). Groundwater well T-2 displayed the highest nitrate concentration. High concentrations of nitrate are likely affected by discharged livestock waste. In this context, coprostanol occurrence showed that the groundwater is polluted by livestock waste. Nitrate concentration in soil was relatively high in samples collected at the LWDS adjacent to the livestock waste treatment plant. Even so, samples from the Nishikawa River Basin displayed higher nitrate concentration in soil as compared to the LWDS samples. In terms of NH_4_^+^, surface water samples collected in the Nishikawa River Basin also displayed nitrate pollution. There have been no remediation activities for the LWDS soil. The area is mainly affected by natural attenuation in soil and groundwater. Thus, the soil is still clearly influenced by animal waste, as indicated by the coprostanol content. Accordingly, the coprostanol content clearly displayed type of soil contamination source. Depending on the location, considering the high concentration of specific ions (SO_4_^2−^, and K^+^) and the detection of coprostanol, the main nitrate sources were shown to be chemical fertilizer in combination with likely influence of livestock waste in the soil and groundwater. Nitrate in groundwater and soil is diluted by precipitation during the rainy season. The dilution is notable only for the unconfined groundwater where nitrate concentration is relatively constant. Nitrate concentration in groundwater is related to the concentration in the surface water both for Nishikawa and Yuegawa River Basins. There is complex but clear relationship between soil and water samples regarding nitrate concentration and high concentration in the soil is likely to be related to the groundwater and surface water pollution. Thus, in addition to the spatial heterogeneity of pyroclastic sediments, nitrate distribution reflects soil, groundwater, and surface water linkage-processes.

The correlation between nitrate and other groundwater chemical components is essential to display the different types of nitrate source. Simultaneous assessment of coprostanol level in soil, surface and groundwater is useful to determine effects of livestock waste and risks for water pollution. In addition, sterol ratio is an effective tool to distinguish between nitrate sources for not only surface water but also groundwater. The relationship between coprostanol (sterol ratio) and nitrate is different depending on the spatial location. Including coprostanol analyses in nitrate assessment is a useful method that can be used in other areas where nitrate pollution sources are investigated.

## Supplementary Information

Supplementary Table S1.
