# Supplementary material for: Spatiotemporal variation of nitrate concentrations in soil and groundwater of an intensely polluted agricultural area
Source: Sci Rep. 2021 Jan 28;11:2598. doi: 10.1038/s41598-021-82188-2 (PMC7844300; doi:10.1038/s41598-021-82188-2)
Supplement: Supplementary file 1 — Supplementary Table S1. [file 41598_2021_82188_MOESM1_ESM.docx]

**Spatiotemporal variation of nitrate concentrations in soil and groundwater of an intensely polluted agricultural area**

**Kei Nakagawa*, Hiroki Amano, Magnus Persson, and Ronny Berndtsson**

Kei Nakagawa (✉)

Institute of Integrated Science and Technology, Nagasaki University,

1-14 Bunkyo-machi, Nagasaki 852-8521, Japan

e-mail: kei-naka@nagasaki-u.ac.jp

Tel.: +81 95 819 2763; fax: +81 95 819 2763

* corresponding author

Hiroki Amano

Department of Kyushu Liberal Arts Education, Tokai University,

9-1-1 Toroku, Higashi-ku, Kumamoto 862-8652, Japan

Magnus Persson

Division of Water Resources Engineering, Lund University,

Box 118, SE-221 00 Lund, Sweden

Ronny Berndtsson

Division of Water Resources Engineering & Center for Middle Eastern Studies, Lund University,

Box 118, SE-221 00 Lund, Sweden

**Supplementary table S1.** Summary of groundwater chemistry and water depth in each well.

| Site | Date | Cl^-^ | NO_3_^-^ | NO_3_-N | SO_4_^2-^ | HCO_3_^-^ | Na^+^ | K^+^ | Mg^2+^ | Ca^2+^ | DO | ORP | EC | pH | Temperature | Coprostanol | Cholestanol | GL- |
| --- | --- | --- | --- | --- | --- | --- | --- | --- | --- | --- | --- | --- | --- | --- | --- | --- | --- | --- |
|  |  | mg L^-1^ | mg L^-1^ | mg L^-1^ | mg L^-1^ | mg L^-1^ | mg L^-1^ | mg L^-1^ | mg L^-1^ | mg L^-1^ | mg L^-1^ | mV | mS m^-1^ |  | ^o^C | ng L^-1^ | ng L^-1^ | m |
| T-1 | Apr 24, 2017 | 11.4 | 42.5 | 9.6 | 30.1 | 31.5 | 8.5 | 22.1 | 6.3 | 23.4 | 9.28 | 376 | 33.5 | 6.03 | 19.7 | 23.8 | 38.6 | 28.1 |
| T-1 | May 11, 2017 | 31.9 | 85.6 | 19.3 | 53.6 | 51.8 | 21.3 | 35.5 | 13.8 | 37.2 | 6.62 | 409 | 48.9 | 6.10 | 18.0 | 20.4 | 51.4 | 31.0 |
| T-1 | Jun 13, 2017 | 29.6 | 81.1 | 18.3 | 51.7 | 59.9 | 21.6 | 29.8 | 14.1 | 39.8 | 8.34 | 373 | 45.2 | 6.23 | 18.5 | 146.0 | 184.0 | 32.0 |
| T-1 | Jul 13, 2017 | 0.9 | 1.1 | 0.3 | 1.1 | 18.4 | 0.4 | 3.2 | 1.0 | 8.0 | 9.21 | 398 | 6.0 | 5.79 | 18.0 | 70.5 | 241.9 | 27.0 |
| T-1 | Aug 8, 2017 | 29.7 | 73.2 | 16.5 | 46.9 | 57.9 | 18.2 | 33.5 | 11.3 | 37.0 | 9.13 | 372 | 43.7 | 6.22 | 18.0 | 7.8 | 34.2 | 32.0 |
| T-1 | Sep 25, 2017 | 17.8 | 60.0 | 13.5 | 44.4 | 46.4 | 15.8 | 39.4 | 9.4 | 22.8 | 10.09 | 354 | 37.9 | 5.95 | 17.5 | 22.4 | 62.5 | 30.0 |
| T-1 | Oct 24, 2017 | 7.4 | 35.5 | 8.0 | 28.0 | 20.3 | 8.7 | 20.1 | 4.5 | 15.2 | 8.94 | 380 | 22.2 | 5.62 | 16.0 | 4.8 | 27.1 | 30.0 |
| T-1 | Nov 20, 2017 | 23.2 | 69.1 | 15.6 | 47.8 | 56.2 | 18.5 | 32.3 | 11.3 | 25.8 | 8.25 | 369 | 42.7 | 6.07 | 16.0 | 3.8 | 23.8 | 28.0 |
| T-1 | Dec 6, 2017 | 27.5 | 76.0 | 17.2 | 51.0 | 59.5 | 23.1 | 29.3 | 14.1 | 32.8 | 9.69 | 380 | 45.1 | 6.05 | 14.0 | 74.6 | 30.5 | 31.0 |
| T-1 | Jan 22, 2018 | 31.0 | 80.8 | 18.3 | 49.1 | 68.5 | 23.0 | 25.3 | 14.1 | 34.3 | 9.90 | 315 | 48.6 | 6.17 | 16.0 | 8.2 | 58.5 | 33.0 |
| T-1 | Feb 26, 2018 | 29.7 | 82.9 | 18.7 | 54.0 | 71.2 | 21.2 | 28.6 | 16.0 | 44.2 | 8.15 | 323 | 50.5 | 6.32 | 16.0 | 82.5 | 173.8 | 35.0 |
| T-1 | Mar 12, 2018 | 19.5 | 64.1 | 14.5 | 46.8 | 64.6 | 16.4 | 37.9 | 12.8 | 31.6 | 10.16 | 355 | 44.2 | 6.18 | 16.5 | 8.2 | 35.5 | 31.0 |
| T-2 | Apr 24, 2017 | 23.1 | 196.0 | 44.3 | N. D. | 42.4 | 16.4 | 9.3 | 19.4 | 55.3 | 9.98 | 333 | 46.4 | 7.83 | 19.0 | 6.0 | 20.0 | 30.0 |
| T-2 | May 11, 2017 | 20.2 | 186.1 | 42.0 | N. D. | 52.1 | 15.0 | 7.6 | 18.8 | 59.2 | 8.68 | 365 | 48.1 | 7.51 | 18.5 | 35.2 | 90.2 | 29.0 |
| T-2 | Jun 13, 2017 | 20.0 | 176.0 | 39.8 | 2.3 | 53.2 | 16.6 | 9.9 | 17.5 | 55.6 | 8.69 | 331 | 45.8 | 7.53 | 19.0 | 24.3 | 67.7 | 30.0 |
| T-2 | Jul 13, 2017 | 19.1 | 174.9 | 39.5 | N. D. | 49.2 | 16.6 | 19.9 | 16.8 | 44.6 | 9.57 | 343 | 48.0 | 7.26 | 18.0 | 18.9 | 48.0 | 30.0 |
| T-2 | Aug 8, 2017 | 20.0 | 174.5 | 39.4 | N. D. | 52.6 | 14.4 | 10.1 | 16.6 | 52.0 | 9.86 | 349 | 46.0 | 7.23 | 17.0 | 16.0 | 37.4 | 32.0 |
| T-2 | Sep 25, 2017 | 18.2 | 156.6 | 35.4 | 2.1 | 52.1 | 16.7 | 10.0 | 16.9 | 45.3 | 10.76 | 323 | 46.6 | 6.83 | 17.0 | 12.1 | 39.6 | 33.0 |
| T-2 | Oct 24, 2017 | 18.5 | 157.0 | 35.5 | 4.0 | 41.2 | 16.4 | 9.9 | 16.8 | 42.7 | 9.10 | 331 | 45.4 | 7.07 | 17.0 | 7.5 | 45.6 | 31.0 |
| T-2 | Nov 20, 2017 | 20.2 | 174.5 | 39.4 | 1.9 | 41.8 | 16.0 | 10.7 | 17.1 | 35.7 | 11.04 | 350 | 45.5 | 6.32 | 16.0 | 1.8 | 27.2 | 31.3 |
| T-2 | Dec 6, 2017 | 18.6 | 159.4 | 36.0 | N. D. | 62.0 | 16.8 | 10.5 | 17.5 | 42.9 | 10.05 | 348 | 46.7 | 6.61 | 14.5 | 51.1 | 49.6 | 31.0 |
| Site | Date | Cl^-^ | NO_3_^-^ | NO_3_-N | SO_4_^2-^ | HCO_3_^-^ | Na^+^ | K^+^ | Mg^2+^ | Ca^2+^ | DO | ORP | EC | pH | Temperature | Coprostanol | Cholestanol | GL- |
|  |  | mg L^-1^ | mg L^-1^ | mg L^-1^ | mg L^-1^ | mg L^-1^ | mg L^-1^ | mg L^-1^ | mg L^-1^ | mg L^-1^ | mg L^-1^ | mV | mS m^-1^ |  | ^o^C | ng L^-1^ | ng L^-1^ | m |
| T-2 | Jan 22, 2018 | 20.7 | 172.3 | 38.9 | 1.7 | 49.6 | 16.8 | 9.5 | 17.8 | 42.4 | 10.48 | 281 | 46.2 | 6.88 | 16.0 | 26.5 | 77.9 | 32.0 |
| T-2 | Feb 26, 2018 | 22.4 | 187.4 | 42.3 | N. D. | 59.0 | 15.4 | 11.0 | 19.4 | 55.1 | 9.19 | 264 | 48.1 | 7.36 | 16.0 | 107.9 | 253.5 | 35.0 |
| T-2 | Mar 12, 2018 | 23.3 | 184.1 | 41.6 | 2.0 | 51.2 | 16.5 | 11.5 | 19.0 | 51.1 | 10.28 | 335 | 48.8 | 6.81 | 16.8 | 52.3 | 125.4 | 31.3 |
| T-3 | Apr 24, 2017 | 14.7 | 35.2 | 7.9 | 10.6 | 66.2 | 12.2 | 5.4 | 9.6 | 25.5 | 9.36 | 355 | 22.4 | 6.84 | 16.0 | 2.8 | 6.4 |  |
| T-3 | May 11, 2017 | 20.6 | 33.4 | 7.5 | 3.4 | 72.3 | 11.0 | 13.7 | 7.9 | 24.4 | 8.53 | 394 | 21.3 | 6.92 | 17.0 | 5.2 | 27.5 |  |
| T-3 | Jun 13, 2017 | 11.0 | 30.7 | 6.9 | 3.8 | 68.7 | 11.0 | 5.2 | 7.2 | 23.5 | 9.19 | 356 | 20.0 | 7.07 | 16.0 | N. D. | 11.9 |  |
| T-3 | Jul 13, 2017 | 11.1 | 30.8 | 7.0 | 3.2 | 74.1 | 11.0 | 4.5 | 7.2 | 24.5 | 9.50 | 365 | 22.5 | 6.88 | 17.0 | 1.9 | 33.5 |  |
| T-3 | Aug 8, 2017 | 11.7 | 32.8 | 7.4 | 3.2 | 78.1 | 12.3 | 7.0 | 8.7 | 25.3 | 8.99 | 371 | 23.1 | 6.99 | 16.0 | 1.2 | 42.4 |  |
| T-3 | Sep 25, 2017 | 11.5 | 30.9 | 7.0 | 6.4 | 83.2 | 12.7 | 6.9 | 8.2 | 24.2 | 9.17 | 346 | 22.2 | 6.86 | 15.5 | 4.7 | 41.8 |  |
| T-3 | Oct 24, 2017 | 11.6 | 30.2 | 6.8 | 6.2 | 76.5 | 12.3 | 6.6 | 8.1 | 23.9 | 9.74 | 344 | 21.4 | 6.71 | 15.0 | N. D. | 37.4 |  |
| T-3 | Nov 20, 2017 | 10.4 | 26.9 | 6.1 | 2.7 | 74.5 | 10.9 | 6.3 | 7.6 | 19.4 | 9.40 | 355 | 19.3 | 6.59 | 15.0 | N. D. | 11.1 |  |
| T-3 | Dec 6, 2017 | 12.2 | 30.1 | 6.8 | 4.2 | 78.2 | 17.4 | 6.5 | 8.5 | 20.9 | 9.84 | 355 | 29.3 | 6.83 | 10.0 | 27.7 | 25.1 |  |
| T-3 | Jan 22, 2018 | 13.0 | 30.2 | 6.8 | 3.7 | 79.4 | 12.7 | 6.1 | 8.9 | 21.0 | 9.25 | 304 | 20.9 | 7.03 | 15.0 | N. D. | 17.9 |  |
| T-3 | Feb 26, 2018 | 11.3 | 31.2 | 7.0 | 4.2 | 69.2 | 10.1 | 6.3 | 7.2 | 20.6 | 8.69 | 275 | 19.9 | 7.47 | 15.0 | N. D. | 0.0 |  |
| T-3 | Mar 12, 2018 | 11.5 | 31.0 | 7.0 | 3.5 | 72.2 | 11.4 | 6.6 | 7.5 | 21.9 | 9.32 | 348 | 20.2 | 6.96 | 15.0 | N. D. | 11.7 |  |
